# Multilocus sequence typing of azole-resistant *Candida auris* strains, South Africa

**DOI:** 10.4102/sajid.v35i1.116

**Published:** 2020-03-23

**Authors:** Rindidzani Magobo, Mabatho Mhlanga, Craig Corcoran, Nelesh P. Govender

**Affiliations:** 1 Centre for Healthcare-associated Infections, Antimicrobial Resistance and Mycoses, Division of the National Health Laboratory Service, National Institute for Communicable Diseases, Johannesburg, South Africa; 2 Ampath National Reference Laboratory, Pretoria, South Africa

**Keywords:** *Candida auris*, azole resistant, multilocus sequence typing, FKS sequencing, South Africa

## Abstract

**Background:**

*Candida auris* is an emerging multidrug-resistant fungal pathogen associated with high mortality.

**Methods:**

We investigated the genetic relatedness of clinical *C. auris* isolates from patients admitted to either public- or private-sector hospitals, which were submitted to a reference laboratory from 2012 to 2015. Patient demographics and clinical details were recorded. We performed antifungal susceptibility testing, sequencing of the hotspot 1 and 2 regions of the *FKS1* and *FKS2* genes for all isolates with an echinocandin minimum inhibitory concentration (MIC) of ≥1 µg/mL and cluster analysis using multilocus sequence typing.

**Results:**

Eighty-five isolates were confirmed as *C. auris*. The median patient age was 59 years [inter-quartile range (IQR): 48–68 years], with male patients accounting for 68% of cases. Specimen types included urine (29%), blood (27%), central venous catheter tips (25%), irrigation fluid (7%), tissue (5%), respiratory tract specimens (4%) and other (3%). Ninety-seven per cent of isolates were resistant to fluconazole, 7% were resistant to both fluconazole and voriconazole, 8% were resistant to both fluconazole and echinocandins (considered multidrug resistant) and all were susceptible to amphotericin B. Of the 15 randomly selected fluconazole-resistant isolates, 14 isolates had an isavuconazole MIC ≤ 1 µg/mL. No FKS mutations were detected. Multilocus sequence typing (MLST) analysis grouped isolates into two clusters: cluster 1 and cluster 2 comprising 83 and 2 isolates, respectively.

**Conclusions:**

Azole-resistant *C. auris* strains circulating in South African hospitals were related by MLST, but the possibility of nosocomial transmission should be explored using a more discriminatory technique, for example, whole genome sequencing.

## Introduction

Since its first description in Japan in 2009, cases of *Candida auris* with varying clinical manifestations have been reported from Asia, Africa, South America, Europe, North America and Australasia.^1,2,3,4,5,6,7,8^ However, the global prevalence and the geographic extent of *C. auris* disease are likely underestimated, especially in the low- and middle-income countries, because conventional laboratory methods misidentify the fungus and relatively few resource-limited countries have the capacity for identification by mass spectrometric or molecular methods.^4,5,6,7^

Of concern, *C. auris* is almost universally resistant to fluconazole, and some isolates have reduced susceptibility to voriconazole, amphotericin B and the echinocandins.^4,6,9^ As expected, persistent or breakthrough fungaemia has been reported among patients initially receiving fluconazole therapy, resulting in death in some cases.^2,4^ *Candida auris* has been associated with large healthcare-associated outbreaks owing to its ability to transmit from person to person, persist for long periods in the hospital environment and resist chemical disinfection by certain products.^10,11,12,13,14^

A whole genome sequencing (WGS) study showed that *C. auris* strains from each of three continents namely, South Asia, South America and Africa, were almost clonal, with simultaneous independent emergence of clonal groups in each continent being hypothesised.^10^ Multilocus sequence typing (MLST) and amplified fragment length polymorphism (AFLP) typing have also demonstrated strain clustering by geographical region.^11,14^ More recently, it has become clear that patients and strains have crossed borders, seeding outbreaks in new locations.^15,16^

In light of an emerging epidemic of *C. auris* infections among hospitalised patients in parts of South Africa, we sought to describe the antifungal susceptibility profile and genetic relatedness of a convenience sample of *C. auris* strains referred to a South African reference laboratory.

## Materials and methods

### Laboratory surveillance

A case was defined as a patient of any age admitted to any South African hospital with first isolation of *C. auris* from any specimen (representing either infection or colonisation) from January 2012 to December 2015. Referring laboratories submitted suspected or confirmed isolates of *C. auris* to the National Institute for Communicable Diseases (NICD) in Johannesburg. Only cases with viable isolates referred to NICD were included in this study. These cases are thus not necessarily representative of all diagnosed cases of *C. auris* over this time period. Corresponding laboratory reports, containing patient demographic information such as age, sex, hospital, specimen type and date of specimen collection were also submitted.

### Species-level identification and antifungal susceptibility testing

Laboratory identification systems regularly misidentify *C. auris* in a predictable way. Referring laboratories, therefore, submitted isolates to the National Mycology Reference Laboratory at NICD when *C. auris* was suspected for species-level confirmation and antifungal susceptibility testing. These were most often yeasts with high fluconazole minimum inhibitory concentrations (MICs) which had been identified as *Candida haemulonii* by Vitek 2 YST using software version 7.0 or earlier versions (bioMérieux, Marcy ľEtoile, France), as *Rhodotorula glutinis* by API 20C Aux or API ID 32C (bioMérieux), as *Saccharomyces cerevisiae* by Auxacolor (BioRad, Hercules, CA, USA) or as *Candida famata* by MicroScan WalkAway (Beckman Coulter, Brea, CA, USA).^17^ Isolates were submitted on sabouraud agar plates or in sealed bottles containing Dorset transport medium [Diagnostic Media Products (DMP), Johannesburg, South Africa] to NICD. We initially performed phenotypic identification using CHROMagar Candida medium (Mast Diagnostics, Merseyside, UK). We subjected all isolates to matrix-assisted laser desorption ionization-time of flight (MALDI-TOF) (Bruker, Bremen, Germany) mass spectrometric analysis using Biotyper v3.1 software (Bruker Ltd., Coventry, UK). Identification of *C. auris* isolates was then confirmed by polymerase chain reaction (PCR) amplification and sequencing of the internal transcribed spacer (ITS) domain of the ribosomal ribonucleic acid (RNA) gene and the D1–D2 region of the 28S subunit using universal primers.^18^ Antifungal susceptibility testing for fluconazole, voriconazole, itraconazole, posaconazole, caspofungin, anidulafungin, micafungin and flucytosine was performed using commercially available microbroth dilution panels containing Alamar blue (Thermo Fisher Scientific, Cleveland, OH, USA). Neither the European Committee on Antifungal Susceptibility Testing (EUCAST) nor the U.S.-based Clinical and Laboratory Standards Institute (CLSI) has defined clinical breakpoints for *C. auris*. For epidemiological purposes, resistance was conservatively defined as follows (adapted from Lockhart et al.^10^): fluconazole ≥ 32 µg/mL, voriconazole ≥ 2 µg/mL (similar to CLSI breakpoints for *Candida krusei*), flucytosine ≥ 128 µg/mL and amphotericin B ≥ 2 µg/mL. Echinocandin resistance was defined using the following tentative MIC breakpoints (adapted from the Centers for Disease Control and Prevention [CDC]^19^): anidulafungin and micafungin ≥ 4 µg/mL. Caspofungin is an unreliable indicator of echinocandin resistance, and caspofungin MICs were not interpreted.^20^ Isavuconazole susceptibility testing was performed on a random sample of 15 fluconazole-resistant isolates using the Etest method. *Candida parapsilosis* ATCC 22019 and *C. krusei* ATCC 6258 strains were included in quality control (QC) runs on all days of testing, and MICs were found to be within the expected QC ranges.

### *FKS* gene sequencing of *C. auris* isolates

We sequenced the hotspot 1 and 2 regions of the *FKS1* and *FKS2* genes for all isolates with an echinocandin MIC of ≥ 1 µg/mL.^21,22^ All sequences were aligned with the FKS sequences of a hypothetical protein obtained from the draft genome sequence of *C. auris* strain VPCI 479/P/13 (accession number CVRJ00000000) from India^23^ and a wild-type *Candida albicans* strain ATCC 90028 (GQ456066).

### Genotyping of *C. auris* isolates

**FIGURE 1:**
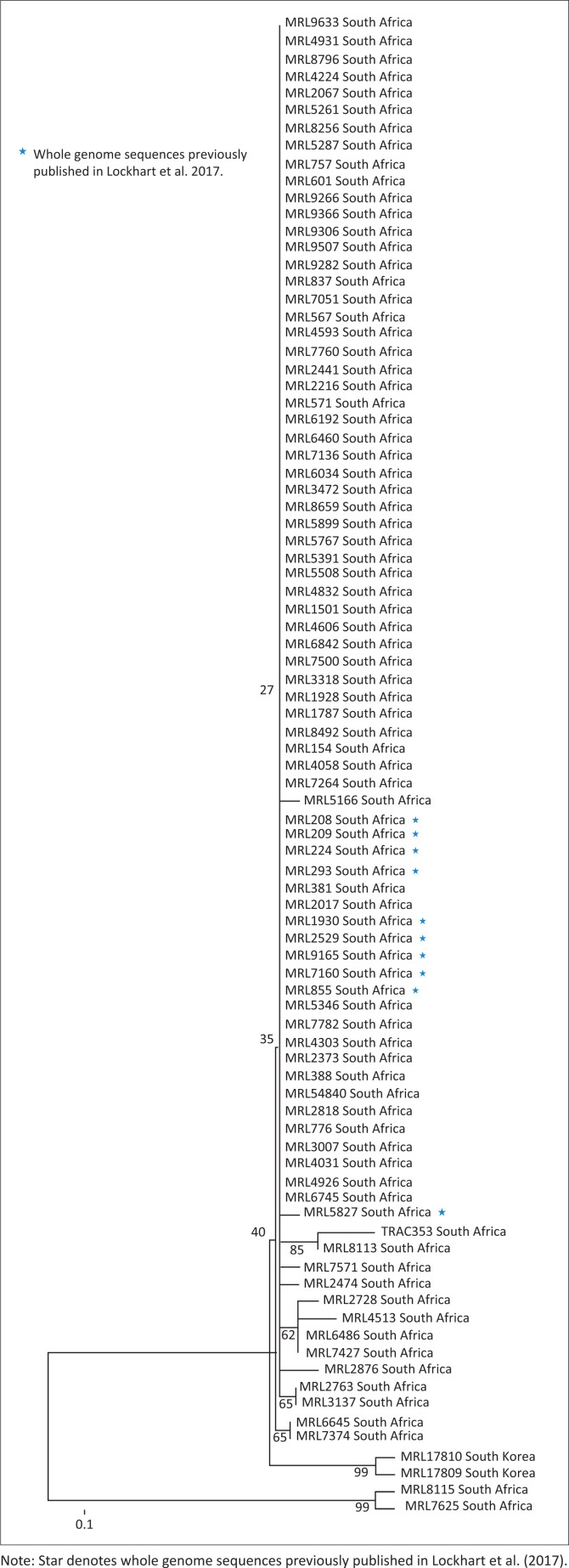
Phylogenetic tree based on multilocus sequence typing (MLST) data of South African *Candida auris* (*n* = 85) isolates using the neighbour-joining analysis method with 2000 bootstrap replications. Sequences of South Korean reference strains (KCTC17809 and KCTC17810) were retrieved from the GenBank.

To determine the genetic relatedness among the *C. auris* strains, we sequenced four loci (ITS, D1-D2, RPB1 and RPB2) using a previously published method.^14^ GenBank accession numbers for the sequence data of ITS, D1-D2, RPB1 and RPB2 loci are attached in the supplementary table. Briefly, we extracted deoxyribonucleic acid (DNA) from single colonies using Zymo ZR fungal/Bacterial DNA MiniPrep (Zymo Research Corporation, Irvine, CA, USA). PCR amplification was performed using primers adopted from Prakath et al.^14^; the following control strains were included: MRL208 (KJ126758) and MRL209 (KJ126759). PCR amplicons were purified using Exonuclease I/Shrimp alkaline phosphatase enzymes (Thermo Fisher Scientific). Deoxyribonucleic acid sequencing was performed with the same primers used for PCR using BigDye terminator version 3.1 in an ABI 3500 genetic analyser (Applied Biosystems, Foster City, CA, USA). Identification of *C. auris* was performed by pairwise sequence comparison of the ITS region using the NCBI BLAST algorithm (www.ncbinlm.nih.gov/blast). We performed multiple-sequence alignment of concatenated sequences using ClustalW.^24^ Using the final MLST data set, we generated a phylogenetic tree by a neighbour-joining statistical method using 2000 bootstrap replications on MEGA 6^25^ (Figure 1). A cluster was defined as isolates from different patients which shared a common ancestor, with a bootstrap value between 90% and 100%.

### Ethical considerations

Ethical clearance to conduct the study was obtained from the Human Research Ethics Committee of the University of the Witwatersrand (Project Research Number: R14/49, Ethical Clearance Number: M140159) on 31 January 2014.

## Results

From January 2012 to December 2015, 86 viable isolates from 77 patients were referred to NICD for identification and antifungal susceptibility testing. One case of *C. haemulonii* infection (1 isolate) was excluded. Cases of *C. auris* infection or colonisation were diagnosed at 32 hospitals, 27 of which were located in Gauteng Province, and most of these were in the City of Johannesburg or Tshwane. The remaining cases were diagnosed at hospitals located in seven other provinces, which included North West, Limpopo, KwaZulu-Natal, Free State, Eastern Cape, Western Cape and Mpumalanga. Seventy-two (85%) isolates were from patients admitted to private-sector hospitals; the remainder were from public-sector hospitals. The median age of cases was 59 years [inter-quartile range (IQR), 48–68 years]. Male patients accounted for 68% (50/74) of the cases. Twenty-nine per cent (22/75) of isolates were cultured from urine followed by 27% (20/75) from blood, 25% (19/75) from central venous catheter tips, 7% (5/75) from irrigation fluid, 5% (4/75) from tissue, 4% (3/75) from respiratory tract specimens and the remainder from miscellaneous sites. Twenty-four (32%) of 76 patients had hospital ward information available. Of these, 12 (57%) were admitted to an intensive care unit, 6 (25%) to a trauma unit, 3 (13%) to a burns unit, 2 (8%) to general wards and 1 (4%) to a neurosurgery ward. No further clinical or outcome information was available.

### Antifungal susceptibility testing

Antifungal susceptibility testing was performed on all 85 *C. auris* isolates (Table 1). Of these, 82 (97%) were resistant to fluconazole with an MIC_50_ and MIC_90_ of 128 µg/mL and 256 µg/mL, respectively. Six (7%) isolates were resistant to voriconazole and seven (8%) isolates were resistant to echinocandins. All six voriconazole-resistant isolates were resistant to fluconazole although none of these were resistant to echinocandins. All seven echinocandin-resistant isolates (8%) were resistant to fluconazole and were thus considered multidrug-resistant, that is, resistant to ≥2 classes of antifungal agents. No isolates were resistant to flucytosine or amphotericin B. Posaconazole and itraconazole had potent in vitro activity with an MIC_50_ and MIC_90_ of 0.25 µg/mL and 0.12 µg/mL, respectively (Table 1). The MIC_50_ (range) for isavuconazole was 0.19 µg/mL (0.06 µg/mL – 4 µg/mL) for 15 fluconazole-resistant strains.

**TABLE 1:** Antifungal susceptibility profile of *Candida auris* isolates (*n* = 85).

Antifungal agent	Number of isolates with MIC (µg/mL) of																Susceptible		Resistant†		MIC_50_ (µg/mL)	MIC_90_ (µg/mL)
	<0.008	0.015	0.03	0.06	0.12	0.25	0.5	1	2	4	8	16	32	64	128	256	*N*	%	*N*	%		
Amphotericin B	-	-	-	-	-	2	42	41	-	-	-	-	-	-	-	-	85	100	0	0	0.5	1
Fluconazole	-	-	-	-	-	-	-	-	-	-		3	5§	23§	27§	27§	3	3	82	97	128	256
Voriconazole	2	6	3	16	24	11	11	6	2§	3§	1§	-	-	-	-	-	79	93	6	7	0.12	1
Posaconazole	38	22	13	6	2	2	1	1	-	-	-	-	-	-	-	-	-		-		0.015	0.06
Itraconazole	-	11	40	23	6	5	-	-	-	-	-	-	-	-	-	-	-		-		0.03	0.12
Isavuconazole‡	-	-	-	1	5	5	2	1		1	-	-	-	-	-	-	-		-		0.19	0.38
Flucytosine	-	1	1	67	11	-	-	-	-	-	-	-	-	5			85	100	0	0	0.06	0.12
Caspofungin	8	-	26	26	6	6		3	3	6	1	-	-	-	-	-	-		-		0.06	2
Anidulafungin	-	-	43	18	4	7	2	8	2	1§	-	-	-	-	-	-	84	99	1	1	0.03	1
Micafungin	-	11	31	21	8	2		1	4	5§	2§	-	-	-	-	-	78	92	7	8	0.06	2

MIC, minimum inhibitory concentration.

† , In the absence of breakpoints, resistance was arbitrarily defined as follows: fluconazole ≥ 32 µg/mL, voriconazole ≥ 2 µg/mL, anidulafungin/ micafungin ≥ 4 µg/mL, flucytosine ≥ 128 µg/mL and amphotericin B ≥ 2 µg/mL;

‡ , *n* = 15;

§ , number of isolates that were resistant.

### *FKS* sequencing

We sequenced the hot spot regions of *FKS1* and *FKS2* genes of 14 isolates with an echinocandin MIC ≥ 1 µg/mL. One isolate was resistant to both anidulafungin and micafungin, and six isolates were resistant to micafungin. No resistance-associated mutations were detected within the hot spot regions of *FKS1* and *FKS2* genes.

### Genotyping of *C. auris* isolates

**FIGURE 2:**
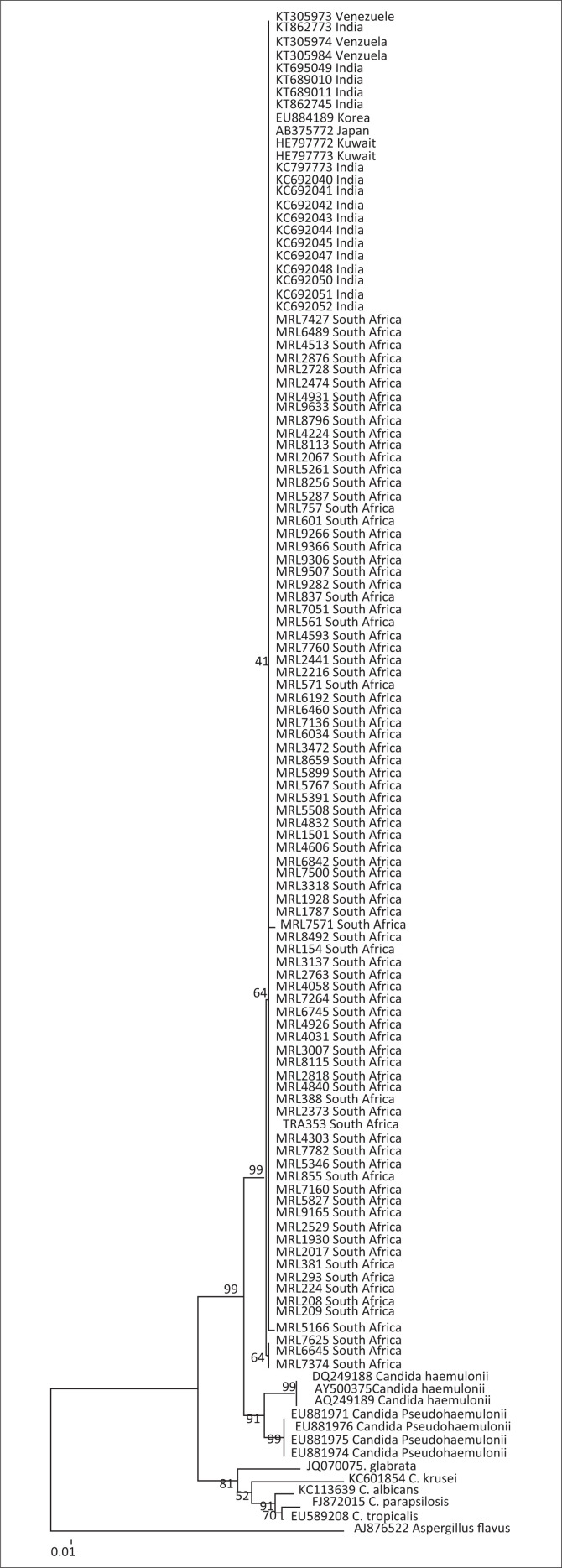
Phylogenetic tree of isolates of the *Candida auris* (*n* = 85) obtained using neighbour-joining phylogenetic analyses and 2000 bootstrap replications based on internal transcribed spacer sequences.

Eighty-five isolates from 76 patients were confirmed as *C. auris* using ITS sequencing. Multilocus sequence typing was performed on all 85 isolates. Phylogenetic analysis grouped the isolates into two clusters, cluster 1 comprising 83 isolates and cluster 2 comprising two isolates (Figure 1). The two isolates belonging to cluster 2 had single-nucleotide polymorphisms (SNPs) within the RPB1 locus, when compared to cluster 1 isolates, and had been cultured from patients at two separate hospitals. Five patients had more than one isolate; isolates cultured from each of these individual patients had similar MLST profiles. A phylogenetic tree based on ITS sequences showed that South African isolates were closely related to Indian and Kuwaiti isolates (Figure 2).

## Discussion

*Candida auris* is an emerging antifungal-resistant pathogen causing outbreaks globally. In South Africa, *C. auris* has reportedly caused large outbreaks in several hospitals, predominantly in private-sector hospitals in Gauteng Province.^26^ Almost all isolates in our case series were resistantw to fluconazole, and a smaller proportion were resistant to both fluconazole and voriconazole or both azole and echinocandin. Despite being cultured from patients admitted to > 30 hospitals, isolates were largely clonal by MLST. A more discriminatory technique, for example, WGS, probably needs to be used to explore isolate relatedness in more detail.

Azole-resistant *C. parapsilosis* is already endemic in private-sector hospitals in Gauteng Province.^27^ We have previously hypothesised that these strains of azole-resistant *C. parapsilosis* initially emerged as a consequence of indiscriminate use of fluconazole for prophylaxis or treatment, and transmission of this pathogen within hospitals then occurred owing to suboptimal adherence to infection prevention and control practices.^27,28^ This is the same setting in which *C. auris*, another azole-resistant pathogen emerged several years ago,^6^ has become endemic and has recently caused several large hospital outbreaks.^26^

Several studies have reported multidrug resistance in *C. auris.*^2,3,4,5,6,7^ Our study confirms that majority of isolates have elevated fluconazole MICs. In a recent WGS study, fluconazole resistance among 10 South African strains was mediated by an F126T substitution in the *ERG11* gene.^10^ The emergence of yet another fluconazole-resistant pathogen in our setting is worrisome, especially in public-sector South African hospitals where echinocandins are available only in tertiary centres. Eight per cent of the studied isolates were resistant to both fluconazole and echinocandin. Multidrug resistance is concerning because this limits treatment to amphotericin B or a combination of antifungal agents. There is no current evidence that strains with elevated echinocandin MICs are associated with clinical failure, with the exception of central nervous system and urinary tract infections (owing to poor penetration of echinocandins into these compartments). Interestingly, we were not able to document any mutations in the *FKS1 or FKS2* genes, even among strains with relatively high echinocandin MICs. Phenotypic echinocandin resistance in these isolates may be mediated by other mechanisms such as enhanced chitin expression and other point mutations outside the hot spot regions of *FKS1* and *FKS2* genes.^29,30,31^ Mutations outside the hot spot regions may have a compensatory effect on the gene leading to alteration in the protein structure that causes variation in the MIC.^30,31^ Echinocandin resistance-associated point mutations have been recently identified in *FKS1 HS1* (S639F/P) of *C. auris* strains exhibiting MIC levels ≥ 4 µg/mL.^20,32,33^ We found some evidence of elevated isavuconazole MICs despite this agent not being available in South Africa. While isavuconazole is efficacious for the treatment of invasive aspergillosis and mucormycosis, there is limited evidence of its efficacy in treating invasive candidiasis.^34^ Five of our tested strains had flucytosine MICs of 64 µg/mL; in contrast, 47% of isolates were flucytosine non-wild type in an Indian study.^9^ This may reflect differing patterns of flucytosine use. Flucytosine is neither registered nor available in South Africa.^35^ Amphotericin B will continue to be recommended as the first-line treatment option for *C. auris* infections in public-sector hospitals where echinocandins are not accessible.^13,36,37^

Combined with the results of classic epidemiologic investigations, molecular data can provide evidence of horizontal transmission in healthcare settings and guide infection prevention and control efforts. Genetic relatedness among *C. auris* isolates has been previously reported. These studies demonstrated that the strains were clonal using pulsed-field gel electrophoresis (PFGE), MLST, AFLP and MALDI-TOF.^11,14^ Another study of isolates from four different countries showed that isolates clustered by geographic regions, when MLST and MALDI-TOF were used. However, South African isolates scattered among different clusters suggesting that different genotypes may be circulating in the country.^14^ Whole genome sequencing of 10 strains of *C. auris* strains from South Africa (strains also included in this study) demonstrated that these strains were highly clonal with a few SNPs (< 70 SNPs).^10^ Using MLST, we have demonstrated that *C. auris* strains are highly related despite being isolated from patients admitted to a large number of hospitals; however, this assay was unable to discriminate strains from different hospitals. This suggests either that clonal strains are circulating or that the method is insufficiently discriminatory. Therefore, a more robust and discriminatory method such as WGS may be needed to demonstrate intra-species variability and nosocomial transmission. Microsatellite genotyping has been used extensively in the detection of *C. parapsilosis* outbreaks in neonatal intensive care units (ICUs) and was shown to be highly discriminatory in uncovering undetected outbreaks.^38,39,40,41^ However, it is difficult to create a microsatellite system for largely clonal pathogen, and this is also a labour-intensive process. Thus, WGS may offer a convenient, although relatively more costly, approach.^10^

This passive surveillance system was limited in several respects. Cases were diagnosed based on clinicians submitting appropriate specimens for fungal culture. Diagnostic laboratories only referred a small proportion of suspected or confirmed *C. auris* isolates to NICD, and referral of suspected antifungal-resistant strains may have biased the reported antifungal susceptibility profile.^26^ NICD’s national active surveillance system for candidaemia (GERMS-SA) will provide a more representative picture of the scale of *C. auris* infection in South Africa.^42^ We envisage undertaking further molecular genotyping using WGS as this is currently the widely used technique to type *C. auris* isolates.

## Conclusion

Azole-resistant *C. auris* strains circulating in Gauteng hospitals were related by MLST but the possibility of nosocomial transmission should be explored using a more discriminatory technique, for example, WGS.
